# Implementing an integrated primary eye care programme in Rwanda

**Published:** 2022-03-01

**Authors:** Ciku Mathenge

**Affiliations:** 1 Professor of Ophthalmology: University of Rwanda and Director of Training and Research: Rwanda International Institute of Ophthalmology, Rwanda.

**Rwanda has implemented a national primary eye care strategy that has seen 2.4 million people screened and over 200,000 referred for eye services at secondary level over the past five years.**

**Figure:**
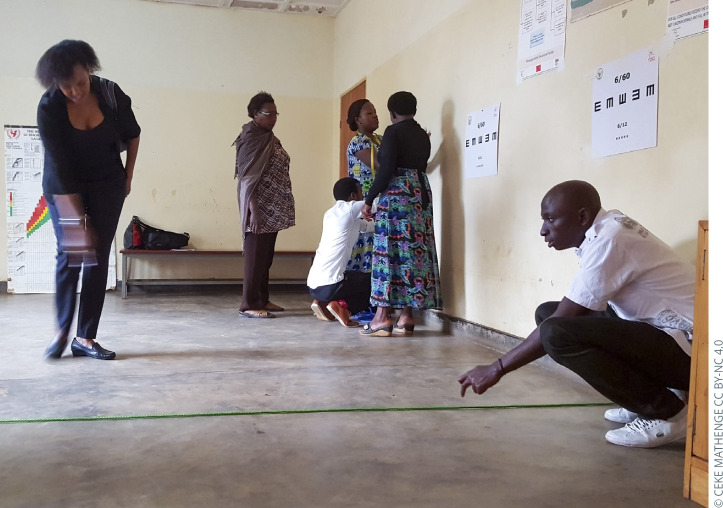
Primary eye care workers are shown how to position visual acuity charts. **RWANDA**

Rwanda is a land-locked country in the Great Rift Valley of Africa and has an estimated population of 13 million. The first prevention of blindness plan in Rwanda was written in 2002. Attempts to implement the plan were hampered by the lack of trained eye health personnel at all levels, which severely reduced the population's access to services. Rwanda's ministry of health, with support from international non-governmental organisations, realised how important it was to establish a primary eye care (PEC) programme for Rwanda that could provide nationwide access to eye care and affordable spectacles for all.

The first attempts to develop PEC programmes were organised as individual projects, run by international non-governmental organisations. A review of the projects concluded that their results were disappointing, as increases in access to care were not maintained.^1^ However, insights from the review were used to develop a new, comprehensive PEC programme which, together with subsequent health system changes, resulted in better organisation of eye care services and strengthened collaboration between stakeholders under the leadership of the ministry of health.

A customised PEC curriculum, aimed at general nurses, was developed based on the same principles, competencies, and protocols as the World Health Organization PEC curriculum for the African region.^2^ Within three years, a dedicated team of five clinical ophthalmic technicians had trained two thousand nurses across all the public primary health care clinics in Rwanda, and the nurses had carried out over 1 million consultations.^3^ By the end of the fifth year, 2.4 million patients had been screened, 2,797 nurses had been trained, and 2,563 nurses had received refresher training. There were also over 200,000 referrals to the secondary level. PEC training has been integrated into the nursing school curriculum, which will provide a sustainable workforce that is less reliant on in-service training.

“PEC training has been integrated into the nursing school curriculum, which will provide a sustainable workforce.”

The key factors for the success of this programme include the following:

1. A very clear and well managed referral pathway from the primary health care facilities to secondary-level hospitals. This ensures that the primary level is not bypassed and the nurses know where to send the patients they cannot manage.^4^
2. High enrolment of the public in community insurance schemes at community level, which ensures that the cost of care is not a barrier.^5^
3. Sustainable supervision of the nurses, thanks to the availability of well-trained allied eye health workers, known as ophthalmic technicians.
4. The high level of engagement by, and support from, the Rwandan Ministry of Health. The ministry of health provided the political leadership and policy influence that allowed system modifications such as changes in the essential drug lists and code of practice of nurses, all of which were needed for the establishment of PEC activities.^6^
5. The development of an ‘Eye Tracker tool’ within the Rwanda integrated health management information system (R-HMIS), which has allowed better monitoring of patient flows.
6. The inclusion of indicators for eye services at the primary level in Rwanda's performance-based health financing scheme – which links payment to outputs or results delivered – has further motivated the nurses to carry out PEC activities.^7^
